# Protoplasm.—Its Relation to Vitality

**Published:** 1874-06

**Authors:** H. R. Noel

**Affiliations:** Professor of Physiology.


### THE AMERICAN JOURNAL OF DENTAL SCIENCE.

**Vol. VIII. THIRD SERIES--JUNE, 1874. No. 2.**

### ARTICLE I.

### *Protoplasm—Its Relation to Vitality.*

BY H. R. NOEL, M. D., PROFESSOR OF PHYSIOLOGY.

**Valedictory Address delivered February 26th, 1874, at Baltimore College of Dental Surgery.**

Within the vast area of scientific investigation, there is perhaps no problem more important than the one known as the "mystery of life." By this we do not mean our ordinary daily lives, our occupations, our pleasures, our sorrows, our successes, our failures, but mean the simple abstract, question life; or that which we possess while the lungs, the heart, and the brain perform their offices, but which ceases when any one of this great "tripod" is stopped.

We associate all ideas of life with the throbbing heart, the expanding lungs, and the thinking brain; we always measure the vitality of any member of the human race by the integrity of these organs, and we look for death or cessation of life by their failure.

But there is a different life and there is a different death going on in our bodies; it begins before our birth and ends after our nominal death, and it is this life and this death underlying the other, measuring and determining the other, which is indeed the great problem to be solved.

To you who have never studied this subject, to you who have never thought of this hidden undercurrent of life, we would address this essay, and use our best endeavor to place in a clear, plain and practical light the knowledge which we professional men think we possess; in other words, we intend giving you a popular lecture upon a professional subject, and we have chosen *life* as the simplest and best as well as most important and most instructive.

You are aware that flowers, grasses, plants, trees, and in fact all varieties of vegetation possess life; you also know that the ox, the horse, the sheep, and other animals which consume these vegetative productions possess life; and we, who eat these animals possess life. So there is something which is common to *plants*, to *animals*, to *man;* did you ever think of it before? Did you ever realize the fact, that you have been so constructed by the author of all being, as to be in certain respects but one whit superior to the flower which blooms in your garden? Certainly it has life and you have life, and most certainly its life and the "underlying life" of which we spoke above, are so nearly allied that to separate them is not in the present state of our knowledge either possible or desirable.

Let us analyze this question of vegetative life, and perhaps we shall thereby learn a useful lesson in regard to the underlying life of human beings; as the two are called by a common name, the analysis of the one may be the solution of the other. In its simplest form this can be done by studying the phenomena in a vegetable cell, for plants, trees, &c., are more or less aggregations of similar parts, and a *cell* bears the same relation to a tree that an individual would bear to a nation. Now, in "cell life," we find in its very lowest forms three elements. 1st. Food and water. 2nd. Light and Heat. 3rd. A worker. The food is carbonic acid, ammonia, &c.; the moisture is the solvent of the food to a great extent, light and heat are supplied by the sun, and the worker combines them. Here we come upon interesting ground, for if the *worker* combines Carbonic Acid, Ammonia, Water, and heat and light, we would like to know something of the manner in which this is done; possibly it is as follows: the worker takes the carbonic acid, and ammonia decomposes them and re-arranges their elements, the carbon or charcoal is retained, the hydrogen and nitrogen are retained, but the oxygen is thrown off and escapes in the air, if not entirely, at least partially. The carbon, hydrogen and nitrogen thus obtained, give material for increase in size, or growth, as we ordinarily say; but besides these we also find that the earth gives certain elements of a mineral character, or saline character, such as soda, potash, lime, iron, &c. Then we must consider the food of a vegetable cell as being supplied to it from the air and from the earth alike; and as plants are aggregations of cells, and even the very largest of trees have the same structure, we are justified in concluding that the history of a *cell* is the epitome of a tree.

What then is the history of this simple cell? Nothing very obscure you would think, yet it is really at this very point that some of the most delicate of all problems arise. A superficial observation would lead us to conclude that a cell would be the easiest thing imaginable to describe and explain; but this is not true, for the *cell* has *life*, takes food and water, heat and light, grows and develops, and could I tell you the philosophy of the life of this *one cell*, I could solve the mystery of life, and in its solution give the explanation of that strange tie which binds man, animals and plants in one common brotherhood.

We can at least, however, describe the phenomena of this life, and this in itself is worthy of most earnest thought, as from this description we deduce conclusions as to our own lives. This *cell* has life, it takes its food and water, it grows, it develops, but in order that it may take its food and water it requires heat and light, and its growth and developement depend entirely upon the heat supplied to it. If these be given in large amounts, the growth is rapid; if in small amounts the growth is slow; here we have upon the very dawn of *life*, upon the very threshold of our subject, two conditions of life—(1) *Material*, (2) *Force*. The food and water are material, the light and heat are forces; and another fact also claims our attention: it is that the material in the cell life, is stored up or deposited in the cells, so that the essence of cell life seems to be increase, accumulation, growth. The carbon, hydrogen, nitrogen and salts, are carefully put away in cells, and so rearranged, so recombined, that we no longer recognized them as distinct elements, but we recognize the *group* as a whole, and call it *wood*, woody fibre, oak, hickory, maple, &c.

Philosophers call the "food and water," "the *material* conditions of life;" they call "light and heat," the "*dynamical conditions of life*," or the forces necessary to life, *i. e.*, vegetative life. But you will perceive that these are only the *conditions*; they are not life itself, but merely the conditions which must envelop something else, and it is this something else which vitalizes the conditions and gives us the phenomena of life.

What is this agent which combines in harmonious union material and force, consumes material and force, and gives us a resultant called growth and development? This is indeed a "worker," this is indeed an architect! This little agent constructs a *cell*, and we say the cell has life; it constructs a plant, and we say the plant has life; millions of them join and construct all the cells of an immense tree, and we say "the tree has life;" really this is not strictly true, for the tree only has life in virtue of the presence of these agents, these factory hands, these workers, which have for their sole object and end, the combination of "material and force"—the union of the ponderable with the imponderable. They construct a "*cell*," or a plant, or a tree, as the coral animalculæ constructs coral reefs, or the sponge animalculæ constructs sponges. This agent has received many names—all, however, suggestive of the one fact, namely, that this is the lowest, the last, and the truly inexplicable origin of vitality; for vitality as seen in plants and animals, is due to the presence of this microscopic agent.

*Protoplasm.*—Huxley calls it protoplasm; Lionel Beale, the great English physiologist, calls it germinal matter; Chambers, of London, calls it "nuclear matter," and all agree that to this agent is to be attributed all of the phenomena of vitality.

When examined under the microscope, it appears to be a glass bead, a spherical speck of jelly, transparent and capable of motion. It is rarely over one thousandth part of an inch in size, and may be much smaller, and yet this microscopic *atom* is the boundary between the physical and the vital; it, and it alone possesses the mysterious power of seizing the elements Carbon, Hydrogen, Nitrogen and Oxygen, &c., and arranging them in the peculiar combinations known as structures ands tisues; it, and it alone possesses the power of catching the light and heat of the sun, and storing it away as it combines the elements, so that a union of the two is effected which cannot be dissolved save by chemcial decomposition. Here at this microscopic atom, all physical philosophers have been arrested in their analyses of the phenomena; here all of our physiologists find the terminus of the path of investigation, and small as it is, atom of jelly as it seems, microscopically minute, almost infinitely little, it holds within itself the mystery of life, and we have hitherto searched in vain for the solution of the pro blem of its weird influence over matter and over force. It is incessantly active, always working, never idle, always building, or if not building, it is crawling about like a snail or leech; possibly inspecting the premises, and noticing all points requiring repairs. You have myriads of them in your blood, myriads in all the tissues of the body, and shrewd microscopists, as Waller, of England; Cohnheim, of Berlin; Stricker, of Vienna; and Dr. Woodward, of Washington; have taken great pleasure in watching the movements of these agents, and speak of their movements from blood vessels to tissues, and among tissues, as *migrations.* In fact, we are now—I mean medical men—in a great controversy as to what is the significance of these very migrations.

This minute agent which moves about and even migrates considerable distances, is found in the simplest of simple forms of vegetation; is found in every plant, in every flower, in every tree; is found in every animal, is found in mankind; and wherever found it has the same appearance, the same form, the same size, the same restless activity.

Let me call your attention to a very important fact in regard to this agent, and the fact is this, that wherever found, whether in plant, animal, or man, it has one and the same office; it has but one occupation, *it is a builder.*

I think we can now understand the value of the word life when applied to plants, animals, and to man alike, or the word vitality when similarly applied. Life, which is thus a common heritage, is due entirely to the presence of this agent, and the *constructive* or *building powers* of this agent give us the phenomena which we term *growth* and *development*, whether seen in a stalk of corn, a young tree, or a growing infant; and in each case and in every case, this constructive factor, this worker called protoplasm, builds the structure, be it vegetable, or be it animal.

Another strange fact is found in this relation of the vegetable and animal kingdoms, and it is an intensely interesting one, that the builders or protoplasm taken from a growing plant, or from the tissues of a human being, are apparently identical in form, size, color, and in every possible physical character, so that no one by a simple examination of any given mass of protoplasm could tell whether it should have the power to build a vegetable tissue, or the power to build an animal tissue; it may have only the power to construct a vegetable cell, or it may have the power to construct a cell of our brain, but whether it possesses one or the other can not be determined by any examination of the little agent itself. To all such interrogatories it returns no answer; it baffles even the most inquisitive microscope, and unless we know its birth-place and home, its special or individual powers are to us a sealed book. And yet in our bodies there are found builders or agents which make only muscle, others which make only nerves, others again are brain builders, and others form our skin, our hair, our nails; but we cannot tell what building powers they have unless we know from what position they were taken, and even then we are liable to make very great mistakes. The mistake may occur from the fact that it is possible that the very builders we have under examination, may not belong in the tissue whence they are taken; they may be out upon a migrating expedition, and having gotten into the blood vessels in a distant part of the body, have been born to this spot in the blood current, and have made a temporary stop before returning home.

From what has been said, you have learned that though we medical men recognize protoplasm as the origin of all vital phenomena connected with growth and development, yet as to the essential nature of protoplasm, as to the inherent powers, and as to why two little agents exactly alike, should have such diverse powers of growth and development, that one shall form bone, the other brain, we are profoundly uninformed. And here the physical philosopher must also stop, for no chemical theories, no theories of physical forces, no theories of electric forces throw one atom of light upon the subject, and we can only say that God in His infinite knowledge has arranged it thus, and placed a barrier beyond which human knowledge has not and probably cannot pass.

But the phenomena of "life," *i. e.*, the observable workings of these agents are open to our investigations, and in this study we have some most instructive acquisitions.

Though vegetable and animal builders are not to be distinguished by their physical characteristics, yet there are certain well marked distinctions in their histories. We find that vegetable protoplasm has the power of combining elements, such as Carbonic Acid, Ammonia, Water, &c., and forming vegetable tissues, fruits, oils, &c.; but the animal agents have no such power; they cannot make tissue out of "inorganic elements," but must have the organic compounds formed by the vegetable agents; hence the food of the vegetable agents is derived from ultimatee lements and simple chemical compounds, but the food of animal builders must be from organized material, must be from either the vegetable structures, or from animal structures; hence all vitality in animals depends upon a prior vitality in vegetables, and the food or materials with which our own tissues are built, have either directly or in directly come to us through the constructive power of vegetable agents. Do not understand that the work performed by the agents in the two kingdoms is different, for it is not; the work is the same, but the material employed for the work is different; the process in each case is of one construction.

*Conditions of Vitality.*—As regards the conditions of vitality, we said that material and force were necessary; in other words, food and water, light and heat; now the protoplasm assimilates or organizes the food, and thus gets material for construction, growth; but at the same time that the material for growth is placed n its proper position, forces are also caught and placed with this material. We cannot destroy matter, we can only change its form. We cannot destroy force, we can only change *one force into another form of force.*

Now vegetation is indirect ratio to heat and light; then we know that heat and light are stimulants to protoplasm; that they are consumed in growth and development; but as they cannot be destroyed, they have only changed form and re appear now as increased vitality in the workers or building agents; but vegetative force, or protoplasmic force, expends itself in construction; where then and how can we find this force thus expended?

In the language of one of England's great philosophers, ("Grove,") "force is continuous and indestructible." Then the physical force *heat*, manipulated or correlated by Protoplasm, re-appears as *vital force*, or as vegetative force, but when this vital force has constructed a tree, for example, an oak, where is the force? If it be continuous and indestructible it has not been lost, and may yet be recovered. The C, H, N, O, &c., material conditions of vitality are found in the bark and wood, a special chemical combination of elements, but where are the heat and light? To say that they are correlated into vitality is no escape from the question, for we instantly ask what has become of the vital force? To this problem there is an easy solution, and it is found in the chemical destruction or decomposition of bark and wood.

If we chemically decompose the oak, there is liberated exactly the amount of heat and light which the sun in years has given to the tree; yes, even the very light which shone upon it in infancy, hundreds of years ago, and the heat which stimulated its growth have been retained, and now in its chemical death are returned.

Chemical death of wood, chemical decomposition, whether rapidly or slowly accomplished, whether by burning or by silent and slow decay, liberates or sets free every particle of force which the protoplasm consumed in the primary construction. Not one *iota* of it is lost; it has been only borrowed, and is now in the final settlement of the account re turned in an exact and just equivalent. The very *gas which*, burning before me to-night, throws its light upon our meeting, was borrowed myriads of years ago from the sun, was caught as light by the protoplasm of that age, was stored away with the material wood; by slow changes this wood has become *coal*, as coal we have received it from the earth, and subjecting it to a partial chemical decomposition in our gas factories, have obtained a portable form of *force* in combination with Carbon and Hydrogen, and when we still further decompose this by process of combustion, we liberate light and heat.

The forces light and heat, *active* or *dynamic*, are rendered *passive* or *static* by protoplasm in the chemical combination of vegetable fibre. So a dynamic division of force is changed into a static condition; and this static condition is again changed into a dynamic by chemical dissolutions? Gun powder and nitro-glycerine are instances of a *static* condition of *force*, and yet readily rendered dynamic by chemical change. A tree is but a static condition of force, and whether it falls in the forest and moulders away by piece-meal for years, or whether it be used by us as fire-wood, in each instance the same amount of force is set free, the same amount of light and heat before static are rendered again dynamic.

You will perceive that the protoplasm in consuming material and force for the growth and development of structures has destroyed neither, but has stored up both; we begin to appreciate the indestructibility of matter and continuity of force, and we look now upon this protoplasmic agent, this indefatigable worker or builder, as being in the vegetable kingdom, the admirable and indispensable manipulator of inorganic elements, and the great preserver of the sun's capital of force loaned to the earth, in the currency of light and heat.

